# Polystyrene microplastics exposition on human placental explants induces time-dependent cytotoxicity, oxidative stress and metabolic alterations

**DOI:** 10.3389/fendo.2024.1481014

**Published:** 2024-11-20

**Authors:** Ashelley Kettyllem Alves de Sousa, Keyla Silva Nobre Pires, Isadora Hart Cavalcante, Iasmin Cristina Lira Cavalcante, Julia Domingues Santos, Maiara Ingrid Cavalcante Queiroz, Ana Catarina Rezende Leite, Alessandre Carmo Crispim, Edmilson Rodrigues da Rocha Junior, Thiago Mendonça Aquino, Rodrigo Barbano Weingrill, Johann Urschitz, Stephanie Ospina-Prieto, Alexandre Urban Borbely

**Affiliations:** ^1^ Cell Biology Laboratory, Institute of Biological and Health Sciences, Federal University of Alagoas, Maceio, Brazil; ^2^ Laboratory of Bioenergetics, Institute of Chemistry and Biotechnology, Federal University of Alagoas, Maceio, Brazil; ^3^ Nucleus of Analysis and Research in Nuclear Magnetic Resonance - NAPRMN, Institute of Chemistry and Biotechnology, Federal University of Alagoas, Maceio, Brazil; ^4^ Institute for Biogenesis Research, Department of Anatomy, Biochemistry and Physiology, John A. Burns School of Medicine, University of Hawaii at Manoa, Honolulu, HI, United States

**Keywords:** metabolomics, microplastics, oxidative stress, placenta, polystyrene

## Abstract

**Introduction:**

Microplastics (MPs) are environmental pollutants that pose potential risks to living organisms. MPs have been shown to accumulate in human organs, including the placenta. In this study, we investigated the biochemical impact of 5 μm polystyrene microplastics (PS-MPs) on term placental chorionic villi explants, focusing on cytotoxicity, oxidative stress, metabolic changes, and the potential for MPs to cross the placental barrier.

**Methods:**

Term placental chorionic explants were cultured for 24 hours with varying concentrations of PS-MPs, with MTT assays used to determine the appropriate concentration for further analysis. Cytotoxicity was assessed using the lactate dehydrogenase (LDH) release assay over a period of up to 72 hours. Reactive oxygen species formation and antioxidant activity were evaluated using biochemical assays. Metabolomic profiling was performed using proton nuclear magnetic resonance (1H NMR).

**Results:**

Placental explants exposed to 100 μg/mL of PS-MPs showed a significant increase in cytotoxicity over time (p < 0.01). Levels of mitochondrial and total superoxide anion (p < 0.01 and p < 0.05, respectively) and hydrogen peroxide (p < 0.001) were significantly elevated. PS-MP exposure resulted in a reduction in total sulfhydryl content (p < 0.05) and the activities of antioxidant enzymes superoxide dismutase (p < 0.01) and catalase (p < 0.05), while glutathione peroxidase activity increased (p < 0.05), and the oxidized/reduced glutathione ratio decreased (p < 0.05). Markers of oxidative damage, such as malondialdehyde and carbonylated proteins, also increased significantly (p < 0.001 and p < 0.01, respectively), confirming oxidative stress. Metabolomic analysis revealed significant differences between control and PS-MP-exposed groups, with reduced levels of alanine, formate, glutaric acid, and maltotriose after PS-MP exposure.

**Discussion:**

This study demonstrates that high concentrations of PS-MPs induce time-dependent cytotoxicity, oxidative stress, and alterations in the TCA cycle, as well as in folate, amino acid, and energy metabolism. These findings highlight the need for further research to clarify the full impact of MP contamination on pregnancy and its implications for future generations.

## 1 Introduction

The annual global production of plastics has reached an unprecedented 400.2 million tons in 2022 (1). This surge in plastic production, coupled with improper waste management, is increasingly contributing to plastic pollution, which is released into natural habitats and leads to significant environmental and potential health risks. Notably, plastic waste constitutes over 75.0% of marine debris, primarily due to its rigid and non-biodegradable nature (2).

The environmental contamination results in the formation of smaller particles known as microplastics (MP), defined as “*synthetic solid particles or polymeric matrices, with regular or irregular shapes and sizes ranging from 1 µm to 5 mm, of either primary or secondary manufacturing origin, and insoluble in water*” (3). The MPs are broadly categorized into two types: primary MPs, which are intentionally incorporated into consumer and commercial products such as cosmetics, microfibers, hygiene products, clothing and fishing nets; and secondary MPs, which arise from the degradation of larger polymers due to physical, chemical, or biological processes in terrestrial or marine environments, such as food packages, water bottles, bags, tire debris etc. (4). Furthermore, MPs can be mainly classified into six categories based on their chemical composition: polyethylene (PE), polystyrene (PS), polypropylene (PP), polyurethane (PU), polyvinyl chloride (PVC), and polyethylene terephthalate (PET) (5). Among these, PS, PET, PE and PP are some of the most widely used plastics and the most common polymers studied (6, 7).

The growing concern over MPs pollution highlights the urgent need for comprehensive research and effective management strategies to mitigate its impact on ecosystems and human health. The life cycle of MPs typically begins with the release of primary or secondary microplastics into the terrestrial and aquatic ecosystems, followed by their transport into water systems (4). Several solutions have been proposed to reduce the amount of such environmental waste. For instance, Osman and colleagues proposed the conversion of PET into solid or liquid fuel, and PET plastic and biomass waste into effective magnetic sorbents for use in waste remediation (7, 8). Such innovations would facilitate the conversion of this abundant waste stream into valuable products, such as energy, while promoting concepts like the circular economy and waste management hierarchy for non-biodegradable waste (7). Another interesting approach is the blending of PS and PU plastic waste into composite resins, which could be reused as binders in the formulation of emulsion paints (9).

Indeed, such innovative ways to deal with MPs waste are much needed, as growing evidence suggest that these MPs can infiltrate the food chain of aquatic organisms and accumulate in their tissues, progressively ascending through the trophic levels (2). This chronic and escalating pollution poses significant threat to all the biodiversity in Earth’s ecosystems, as MPs have been shown to not only bioaccumulate, but also impact a wide variety of living organisms, including animals (10, 11). The MPs have been identified in both invertebrates and vertebrates, not only within their immune and digestive systems but also accumulating in their tissues and fluids. In addition to the MPs retention in their tissues, adverse health effects and even mortality have been reported to a variety of animals (12, 13).

In humans, the placenta was the first organ to be reported with MPs presence (14, 15). Afterwards, several other studies have documented the presence of MPs in placentas worldwide (16–23), and in other human organs (24–28) Nevertheless, significant gaps in the data regarding exposure and hazard remain, necessitating further investigation. Consequently, it is currently not possible to perform a human health risk assessment (HRA) for contamination by plastic pollutants (29). Although HRA for the MPs are still debatable, the toxicity on human health caused by MPs may be due to the nature of plastic; such is the case of styrene-butadiene rubber (30); the other one is the size of the particles (31).

Such growing evidence from diverse research groups worldwide supports the idea that MPs not only accumulate in placentas but also indicates the possibility that they could affect the fetal development. In the absence of large epidemiological studies, both *in vivo* and *in vitro* studies can delineate the molecular initiating event and subsequent key pathways that lead to potential adverse outcomes (32).

Experimental *in vivo* models of MPs exposure during pregnancy have demonstrated increased cytotoxicity, oxidative stress, inflammation, and cognitive and neurological changes in the offspring (33–36), as well as reproductive toxicity (37). *In vitro*, studies using placental cell lines, such as HTR-8/SVneo, Bewo and Swan 71 cells have demonstrated that PS MPs can be internalized and change certain cell functions, which in turn, would alter the placenta physiology (38–40). Advancing research in this area with primary placental cells or tissues is crucial for assessing the potential hazards and risks of MPs exposure to maternal and fetal health (41). Therefore, our study aims to determine whether exposure to MPs on term placental chorionic villi explants can induce biochemical and metabolic alterations in the placenta.

## 2 Materials and methods

### 2.1 Sample collection

Term placentas were obtained from a cohort of 40 anonymous healthy pregnant women (average age of 25.9 ± 5.2 years old), carefully matched for gestational delivery week (37.8 ± 2.6 weeks), who provided consent following uncomplicated pregnancies and underwent caesarean section deliveries. Noteworthy, these placentas were not analyzed for MP they eventually could already contain. Ethical approval for research involving human subjects was obtained from the Ethics Committee (CAAE 58129422.3.0000.5013). Sampling took place at Hospital Universitario Professor Alberto Antunes (HUPAA) of the Federal University of Alagoas (UFAL) between February 2023 and September 2023. Exclusion criteria encompassed gestational diabetes, gestational hypertensive diseases, chorioamnionitis, membrane rupture, and any other maternal medical conditions, as well as known fetal aneuploidy, fetal growth restriction, and macrosomia.

### 2.2 Term chorionic villi explant culture

The placental explants were obtained following a protocol based on the methods initially developed by Trowell (42), with current modifications implemented and extensively published (43). Cotyledon fragments were thoroughly rinsed in glass-filtered phosphate buffer solution (PBS, 1.6 μm Whatman GF/A, Sigma-Aldrich, St. Louis, MO, USA) to remove maternal blood and to avoid baseline plastic exposure or any contamination by MP. Only the villous tissue was minced with a number 21 scalpel, generating approximately 5 mg of tissue per well. These placental explants were cultured in individual glass Petri dishes in DMEM/F12 culture medium (Sigma-Aldrich) with 10% fetal bovine serum (FBS), 1% penicillin/streptomycin and 1% gentamicin (Sigma-Aldrich), and incubated at 37° C and 5% CO2. To prevent contamination from laboratory-derived plastics, all laboratory materials, typically constituted by plastic, were substituted for glass materials. Whenever a plastic substitute could not be employed, this limitation was addressed in the text.

### 2.3 Polystyrene microplastic exposure to placental explants

Polystyrene microplastics (PS-MPs) of 5 μm functionalized with carboxylic surface groups were used (Magsphere, Los Angeles, CA, USA). Thus, the structure and composition of polystyrene were corroborated using Raman spectroscopy (XploRA Raman spectrometry; spectral range 300–2200 cm−1, 532 and 785 nm laser diodes, 600 lines per mm grating, Horiba, Japan) and analyzed with KnowItAll software (Wiley Science Solutions, Hoboken, NJ, USA), and the free microplastic libraries SLOPP and SLOPPe (**Supplementary Figure 1**). No pre- or post-treatments were performed in the PS-MPs. Exposure time and concentration were defined in subsequent experiments, also based on the results reported in literature (44). Bisphenol A (BPA) (Sigma-Aldritch) was employed as positive control for oxidative stress experiments at the concentration of 1 μM according to the literature (45).

### 2.4 MTT assay

Placental explants were assessed using the 3-(4,5-dimethylthiazol-2-yl)-2,5-ditetrazolium bromide (MTT) assay, following the manufacturer´s protocol (INLAB, SP, Brazil). Explants were treated with PS-MP concentrations of 0.1, 1, 10, and 100 µg/mL (w/v) for 24 h to establish an experimental non-lethal concentration of PS-MP. Briefly, placental explants were transferred to a 96-well plastic plate, and incubated for 4 h at 37°C with 5 mg/mL of MTT in DMEM/F12 (Sigma-Aldrich). Following PS incubation, the absorbance was measured at 540 nm using a Bio-Rad Multiwell Plate reader (Bio-Rad, Hercules, CA, USA). All experiments were performed in triplicates (n=3).

### 2.5 LDH release assay in placental explants

PS-MP cytotoxicity was measured using the Pierce LDH Cytotoxicity Assay Kit, according to the manufacturer’s instructions (Thermo Fisher Scientific, Waltham, MA, USA). Briefly, placental explants were treated or not with PS-MP for 24, 48 and 72 h for a time-dependent cytotoxicity assessment. Following PS-MP exposure, 50 μL of conditioned medium from each sample were transferred to a 96-well plastic plate, and 50 μL of Reaction Mixture were added. After 30 minutes, protected from light, 50 μL of Stop Solution were added to each well, and the absorbance was measured at 490 and 680 nm on a Bio-Rad Multiwell Plate reader (Bio-Rad). To calculate the percentage of cytotoxicity, the LDH activity of the spontaneous LDH Release Control (water-treated) was subtracted from the PS-treated sample LDH activity and divided by the total LDH activity [(Maximum LDH Release Control activity) – (Spontaneous LDH Release Control activity)], and multiplied by 100, according to the manufacturer. The experiments were made in triplicates, and the cytotoxicity for each n was calculated (n=3). Noteworthy, we have accompanied the control group dynamics of LDH release through time, to make sure that the placental explants were not naturally dying over time, and up to 72 h of culture they were considered viable (Average O.D. of 1.22 at 24 h, 1.3 at 48 h, and 1.529 at 72 h, while the maximun LDH release positive control had an average O.D. of 2.23 at 24 h, 2.3 at 48 h, and 2.46 at 72 h).

### 2.6 Oxygen superoxide production

The O2•– production was evaluated by two methods. The first method focused on the overall production of O2•– by nitroblue tetrazolium (NBT) (Sigma-Aldritch) (46). After 1 h of incubation, a solution of 1 M KOH and DMSO (v/v) was added, and the placental explants were transferred to a 96-well plastic plate. The absorbance was measured at 630 nm using a spectrophotometer (AJX-6100PC, Micronal). The second method was performed to access only the mitochondrial O2•– production, through mitoSOX staining (47) at 5 µM at an excitation wavelength (λex) of 510 nm, emission wavelength (λem) of 580 nm, and a 10 nm slit width for both excitation and emission. (Invitrogen, Waltham, MA, USA), and according to the manufacturer’s instructions. The fluorescence was measured with two probes (Invitrogen) and a spectrofluorimeter (Shimadzu RF5301, Shimadzu, Columbia, MD, USA). All experiments were conducted in triplicates (n=3).

### 2.7 Hydrogen peroxide levels

The production of H_2_O_2_ was monitored using the fluorescent probe Amplex Red (10 μM, Thermo Fisher Scientific) (48). This experiment was conducted with peroxidase (1 U/mL) at an excitation wavelength (λex) of 563 nm, emission wavelength (λem) of 587 nm, and a 3 nm slit width for both excitation and emission. The H_2_O_2_ was expressed in µmol/mg of protein-1 and rendering to a calibration curve. All experiments were conducted in triplicates (n=8).

### 2.8 Superoxide dismutase activity

The placental explants homogenate (80 μg protein) were incubated in 50 mM sodium carbonate buffer (NaCO_3_/NaHCO_3_ at pH = 10.2 with 0.1 mM EDTA) at 37°C. The reaction was initiated by adding 20 μL epinephrine (150 mM) in acetic acid (0.05% v/v). The absorbance was measured at 480 nm with a dual-beam scanning spectrophotometer (AJX-6100PC, Micronal) for 5 min. One SOD unit was defined as the amount of protein to inhibit the autoxidation of epinephrine (1 μM) per minute. The results were expressed as U mg-1 protein (49). All experiments were conducted in triplicates (n=5).

### 2.9 Catalase activity

To determine the decomposition of H_2_O_2_ in water and oxygen, 40 μg equivalent of protein (from the placental explants homogenate) was added to 50 mM phosphate buffer (KH_2_PO_4_ + Na_2_HPO_4_ with pH = 7.0) at 25°C. The reaction was started by adding 10 mM of H2O2. The analytical signal decrease was monitored at 240 nm with a dual-beam scanning spectrophotometer (AJX-6100PC, Micronal) for 2 min. One unit of CAT was defined as the amount of protein required to convert 1 μM of H2O2 to H_2_O and O_2_ per minute (50). The results were expressed as K mg-1 protein and all experiments were conducted in triplicates (n=5).

### 2.10 Glutathione peroxidase activity

The GPx activity was monitored by determining the decrease in nicotinamide adenine dinucleotide phosphate (NADPH) analytical signal at 340 nm using a scanning spectrophotometer (AJX-6100PC, Micronal) for 3 min at 20°C (51). The mixture contained 80 μg protein (from the placental explants homogenate), 0.05 M Na2HPO4 (pH = 7.0), 5 mM EDTA, 84 μM NADPH, 1.1 mM sodium azide, 1.5 mM of GSH, 0.1 U of GR, and 90 μM of H2O2. One enzyme unit was defined as the amount required for the oxidation of 1 μM of NADPH min-1 mg-1 of protein. The results were expressed as U mg-1 protein and all experiments were conducted in triplicates (n=5).

### 2.11 Quantification of the redox state by reduced glutathione and oxidized gluthathione relation

The GSH/GSSG ratio was assessed by measuring the levels of GSH and GSSG (52). The homogenates containing 50 μg and 100 μg of protein and o-phthalaldehyde (1 mg mL-1) were utilized. For the GSH assay, 0.1 M NaH2PO4 (pH 8.0) and 0.005 M EDTA were used as buffer, whereas 0.1 M NaOH was the buffer used for the GSSG reaction. Fluorescence was monitored using a spectrofluorometer (Flex Station 3, Molecular Devices LLC, San Jose, CA, USA) with an excitation wavelength (λex) of 350 nm and an emission wavelength (λem) of 420 nm. An analytical curve based on known concentrations of GSH and GSSG was used to determine the concentrations of these compounds in the samples. All experiments were conducted in triplicates (n=5).

### 2.12 Sulfhydryl group content

Total protein quantification of the placental explants was conducted using the Bradford method, and a BSA solution (3 mg mL-1) was used as a standard for total protein determination. The total SH content in the samples was determined based on the reaction with 5,5’ Dithiobis (2 Nitrobenzoic Acid) (DTNB, Sigma-Aldritch). The homogenate (100 μg protein) was incubated in the dark with DTNB (500 μM) for 30 min. Afterwards, the final volume was adjusted with extraction buffer (50 mM Tris-HCl with 1.0 mM EDTA, pH 7.4), and the absorbance was measured at 412 nm with a dual-beam scanning spectrophotometer (AJX-6100PC, Micronal, Sao Paulo, Brazil) (53). All experiments were conducted in triplicates (n=5).

### 2.13 Malondialdehyde content

A total of 450 μg of tissue homogenate protein was combined with 200 μL of 30% (v/v) trichloroacetic acid (TCA). Subsequently, Tris-HCl (10 mM) was added and centrifuged at 1180 g for 10 min at 4°C. Then, the collected supernatant was mixed with 0.73% (m/v) thiobarbituric acid, which reacted with lipid peroxidation products to form a pink-colored compound. Following this, the mixture was incubated for 15 min at 100°C and cooled down. The absorbance was measured using a spectrophotometer (AJX-6100PC, Micronal) at 535 nm. The results were expressed in mmol mg-1 of protein (54). All experiments were conducted in triplicates (n=5).

### 2.14 Protein carbonylation

The quantification of carbonyl group formation in 100 µL of tissue homogenate was conducted using the reaction with 2,4-dinitrophenylhydrazine (DNPH) (55). The reaction control was treated with 2.5 M HCl, whereas the samples groups were treated with 10 mM DNPH (diluted in 2.5 M HCl). Subsequent to room temperature incubation, 2 M trichloroacetic acid (TCA) was added, followed by centrifugation at 10,000 g for 10 min at 4°C. After sequential steps of centrifugation and washing, an ethanol and ethyl acetate mixture (1:1 v/v) was added. The groups were finally suspended in 6 M guanidine and centrifuged again. The collected supernatant was used for absorbance measurement at 370 nm using a plate reader, normalized to the protein content (Tecan Infinite 200 pro, Männerdorf, Switzerland). The results were expressed in pmol mg-1 protein. All experiments were conducted in triplicates (n=5).

### 2.15 Proton nuclear magnetic resonance-based metabolomics

The supernatants of 72 h explant cultures were prepared by adding 350 μl of 0.1 M phosphate buffer containing 3-[Trimethylsilyl] propionic-2,2,3,3-d4 acid sodium salt at 0.1 mM in D2O. The prepared samples were then transferred to 5 mm NMR tubes. All 1H NMR spectra were acquired on a Bruker 600 MHz spectrometer Avance III (Bruker BioSpin, Billerica, MA, USA) equipped with a PA BBO 600 S3 BBF-H-D-05 Z SP probe. Before the experiments, the NMR spectrometer was calibrated daily following strict standard operating procedures to ensure the highest spectral quality and reproducibility. A sealed tube containing a 99.8% MeOD standard sample was used to ensure that supernatants were run at 298 K. The ‘edte’ command controlled the temperature’s stability over time. The second calibration with sucrose aimed to optimize water suppression, and spectrometer sensitivity. An automatic tune and matching procedure, deuterium signal lock, and automatic shimming routine were performed before each experiment. The NMR spectra were recorded using the 1D NOESY pulse sequence with water suppression (pulse program: noesygppr1d). For all experiments, 128 scans were recorded with 32 k data points, a spectral width of 20.029 ppm, and a relaxation delay of 4 s. The spectral processing was realized automatically using TopSpin 3.5 (Bruker BioSpin, Billerica, MS, United States), performing phase and baseline correction, and calibrating the TSP reference signal at δ0.00 ppm. The peaks were identified using the Human Metabolome Database (HMDB) (The Metabolomics Innovation Centre, Canada) and the ChenomX NMR Suite (Chenomx Inc., Canada). Criteria such as chemical shifts and multiplicity were employed for peak identification. The dataset underwent further processing using the R statistical software, version 4.1, along with the R package PepsNMR (R Core Team, Vienna, Austria). The spectral region spanning from 4.7 to 5.1 ppm, which includes the residual water signal, was selectively suppressed. Biomarker discovery and classification were performed using the online platform MetaboAnalyst version 6 (www.metaboanalyst.ca).

The supervised model Orthogonal Partial Least Squares Discriminant Analysis (OPLS-DA) was executed to evaluate the capacity of metabolomics data in distinguishing PS-MP exposed samples from control samples, considering the complete set of identified metabolites. The defined confidence region was set to 95%, and one hundred permutations were employed for model evaluation. Significant metabolites (biomarkers) were selected by analyzing loadings and variable importance in the projection (VIP) plot from the OPLS-DA model, indicating the variables crucial for group discrimination. Additionally, a heatmap analysis was applied to reveal metabolic patterns between the groups and a t-test with false discovery rate (FDR)-corrected p values was employed to assess the significance of metabolites. The metabolomics biomarkers data were correlated with oxidative stress parameters. Finally, to estimate the association between the two datasets, it was applied the Pearson correlation coefficient and a multiblock Partial Least Squares Discriminant Analysis (PLS-DA) model, also known as Data Integration Analysis for Biomarker Discovery using Latent Components (DIABLO) (56). All experiments were conducted in triplicates (n=16).

### 2.16 Zeta potential

The 100 μg/mL PS-MP in DMEM/F12 culture media had their zeta potential measured by a nano/zeta sizer ZS90 (Malvern Instruments Ltd) with 250 scans made in five different samples.

### 2.17 Statistical analysis

With the exception of metabolomics already described, the statistical analyses was performed using the one-way ANOVA followed by Bonferroni multiple comparisons. All the statistical analyses were performed using GraphPad (version 7.0; GraphPadSoftware, Inc.), with p < 0.05 considered statistically significant.

## 3 Results

### 3.1 Polystyrene exposition is cytotoxic for placental explants

**Figure 1:**
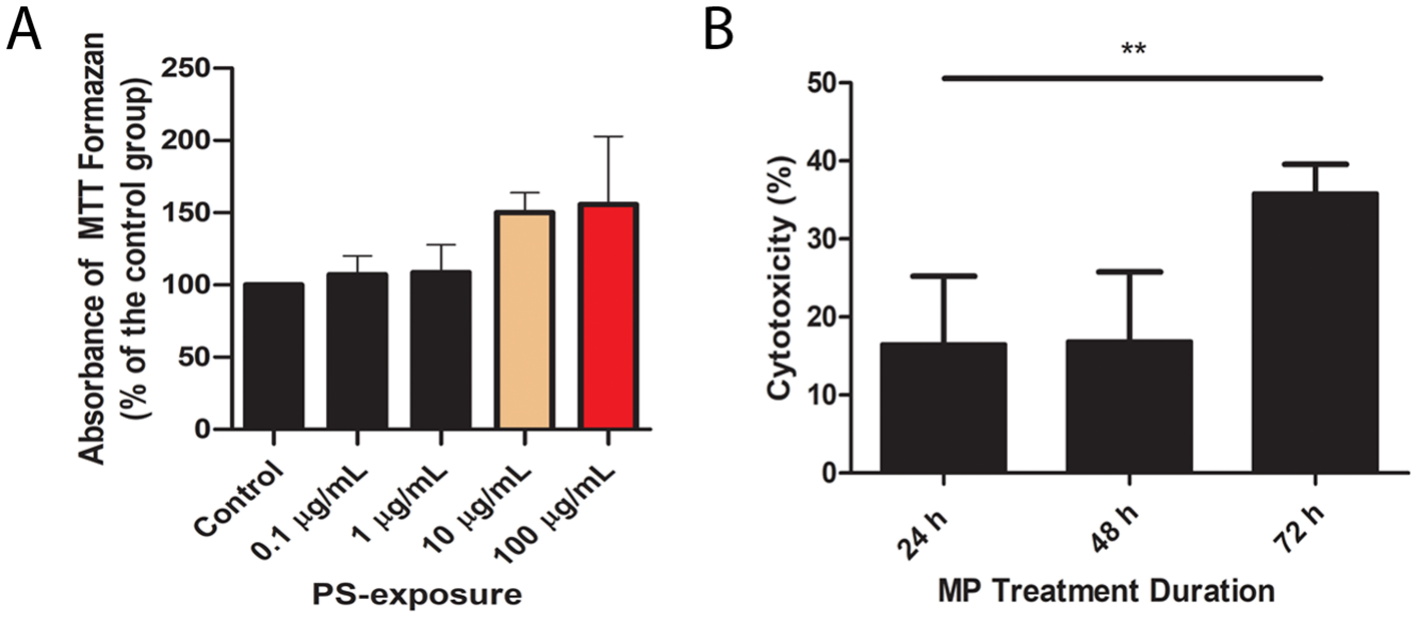
Polystyrene exposure induces cytotoxicity in placental explants, as measured by metabolic activity and membrane integrity. **(A)** MTT assay results show the effects of increasing PS concentrations (0.1, 1, 10, and 100 μg/mL) over 24 h, indicating reduced metabolic activity. **(B)** LDH release assay evaluates membrane damage following exposure to 100 μg/mL PS over 24, 48, and 72 (h) Experiments were conducted in triplicate (n=3) with results analyzed via one-way ANOVA and Bonferroni correction. **p < 0.01 compared to control.

Exposure to different concentrations of PS-MP for 24 h did not affect explant viability as determined by the screening MTT test (**Figure 1A**). Based on these results of viability, the concentration of PS-MP to be tested in all further experiments were 100 μg/mL, as it was not only inducing viability reduction, but the increased tendency observed caught our attention.

To assess if such concentration would present time-dependent cytotoxicity, the LDH release assay was performed after exposure of the placental explants for 24, 48 and 72 h. The cytotoxicity after 24 h was 10.37 ± 3.79%, after 48 h was 23.19 ± 3.09%, and after 72 h was 40.32 ± 7.02%. The 30% increase in cytotoxicity from 24 to 72 h was statistically significant (p < 0.01) (**Figure 1B**).

### 3.2 Polystyrene exposition leads to oxidative stress on placental explants

**Figure 2:**
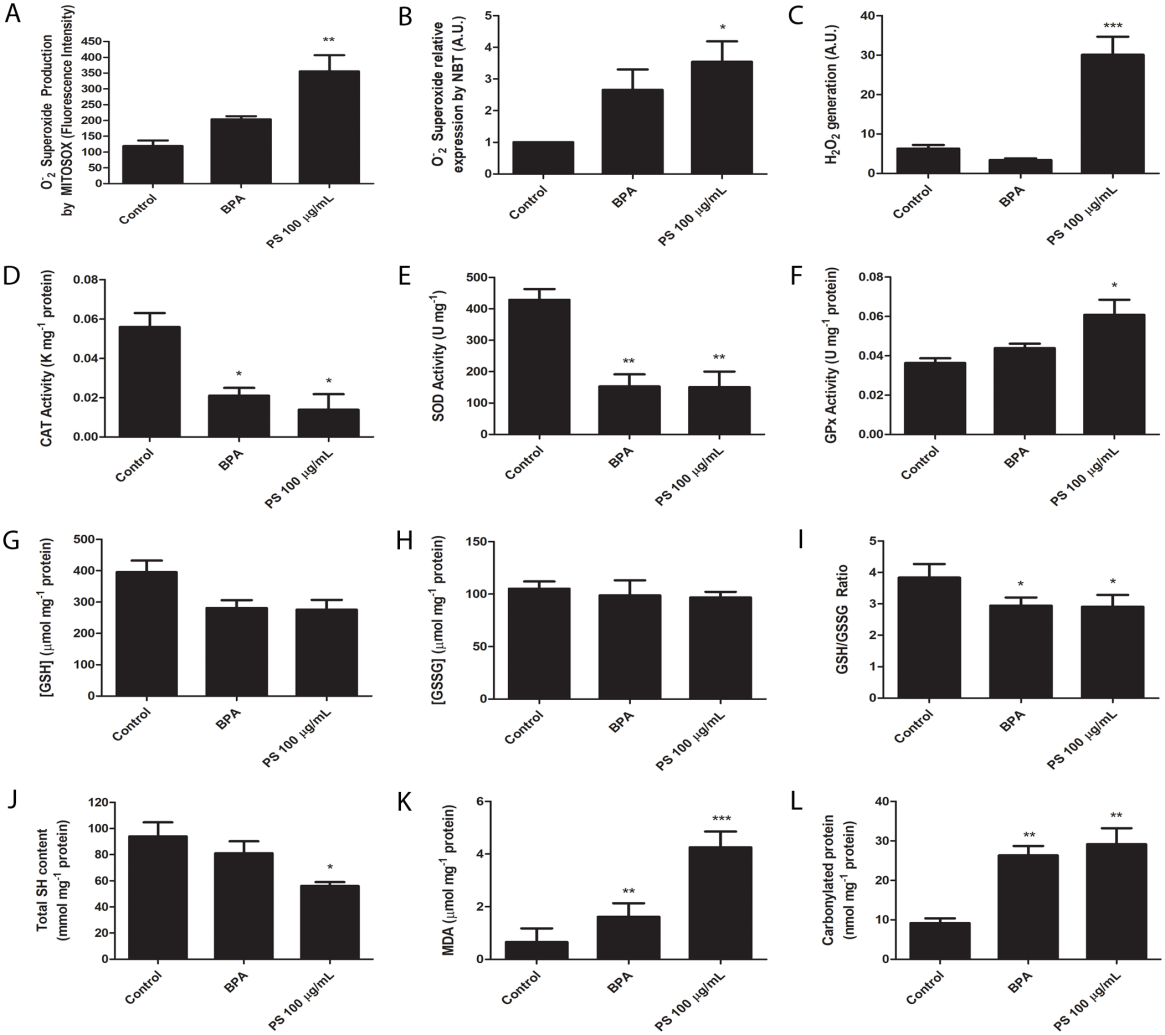
Placental explants exposed to 100 μg/mL PS microplastics or 1 μM bisphenol A (BPA, positive control) exhibit changes in oxidative stress markers. **(A)** Oxygen superoxide (O_2_•–) production by mitochondria, **(B)** O_2_•– total amount, **(C)** hydrogen peroxide (H_2_O_2_) production, **(D)** catalase (CAT) activity, **(E)** superoxide dismutase (SOD) activity, **(F)** glutathione peroxidase (GPx) activity, **(G)** reduced glutathione ([GSH]) levels, **(H)** oxidized gluthathione ([GSSG]) levels, **(I)** GSH/GSSG ratio, **(J)** total sulfhydryl (SH) content, **(K)** malondealdehyde (MDA) levels, and **(L)** and carbonylated protein levels. All experiments were conducted in triplicates, and n=3 for MitoSOX analysis, n=8 for H_2_O_2_ analysis, and n=5 for the other experiments. One-way ANOVA followed by Bonferroni multiple comparisons was used for all experiments. *p < 0.05, **p < 0.01, ***p < 0.001 compared to control.

As MP-PS exposure showed time-dependent cytotoxicity to placental explants, we evaluated if they would trigger an oxidative stress response. We assessed the production of mitochondrial O_2_•– and overall O_2_•– levels in placental explants using MitoSOX staining and the NBT assay, respectively. Both methods demonstrated a significant increase in O_2_•– levels, with mitochondrial O_2_•– increasing by 3.5-fold and total O_2_•– showing a similar elevation upon exposure to PS-MP (p < 0.01 and p < 0.05, respectively) (**Figures 2A, B**). The similar pattern was observed in the analysis of H_2_O_2_ generation, which greatly increased from 6.29 ± 0.43 A.U. to 30.10 ± 0.43 A.U. (p < 0.001) (**Figure 2C**). The activity of antioxidant enzymes was also assessed. SOD catalyzes O_2_•– in O_2_ and H_2_O_2_, helping to regulate mitochondrial function as it scavenges O_2_•–, produces H_2_O_2_ and inhibits ONOO- production by protecting NO, while CAT and GPx are responsible of breaking H_2_O_2_ in O_2_ and H_2_O. We observed a great reduction of SOD and CAT activity when explants were exposed to PS, as CAT was reduced from 0.056 ± 0.007 K mg-1 protein in the control group to 0.014 ± 0.008 K mg-1 protein in the PS group (p < 0.05) (**Figure 2D**). Similarly, SOD was reduced from 428.5 ± 34.58 U mg-1 protein in the control group to 150.4 ± 49.84 U mg-1 protein in the PS group (p < 0.01) (**Figure 2E**). In contrast, GPx activity was increased from 0.036 ± 0.002 U mg-1 protein in the control group to 0.061 ± 0.008 U mg-1 protein (p < 0.05) (**Figure 2F**).

Since GPx activity was increased, we evaluated GSH and GSSG levels. While GSH had a tendency of reduction in the PS group (**Figure 2G**), and GSSG was unchanged (**Figure 2H**), their ratio was reduced in the PS group, from 3.84 ± 0.43 to 2.91 ± 0.38 (p < 0.05) in the control group (**Figure 2I**). Altogether, these results indicate the presence of oxidative stress due to reduced SOD and CAT capabilities, and a reduced GSH/GSSG ratio coupled with increased GPx activity indicates this antioxidant defense system is actively engaged in counteracting the oxidative stress.

Another well-known marker of oxidative stress is the total SH content, which prevent uncontrolled oxidation of proteins by Cystein aminoacids. We observed a clear reduction of the total SH content, from 93.93 ± 10.84 mmol mg-1 protein in the control group to 56.05 ± 2.96 mmol mg-1 protein in the PS exposed group (p < 0.05) (**Figure 2J**).

Due to the observation that exposure to PS-MP results in an increase in all reactive oxygen species (ROS), it is crucial to comprehend the extensive oxidative stress experienced by placental explants. Therefore, we conducted further analyses on ROS production indicators, including measurements of malondialdehyde (MDA) and carbonylated protein levels. MDA is one of the final products of polyunsaturated fatty acids peroxidation, while carbonylation of proteins is defined as the covalent, nonreversible modification of the side chains of cysteine, histidine and lysine residues by lipid peroxidation end products, resulting in their loss of function. Our results showed that MDA level substantially increased, from 0.66 ± 0.52 μM mg-1 protein in the control group to 4.26 ± 0.60 μM mg-1 protein in the PS group (p < 0.001) (**Figure 2K**). This elevation was concomitant with a rise in protein carbonylation, escalating from 9.18 ± 1.20 nmol mg-1 protein in the control group to 29.16 ± 4.06 nmol mg-1 protein in the PS group (p < 0.01) (**Figure 2L**).

### 3.3 Placental chorionic villi metabolism is disrupted by PS-MP exposure

**Figure 3:**
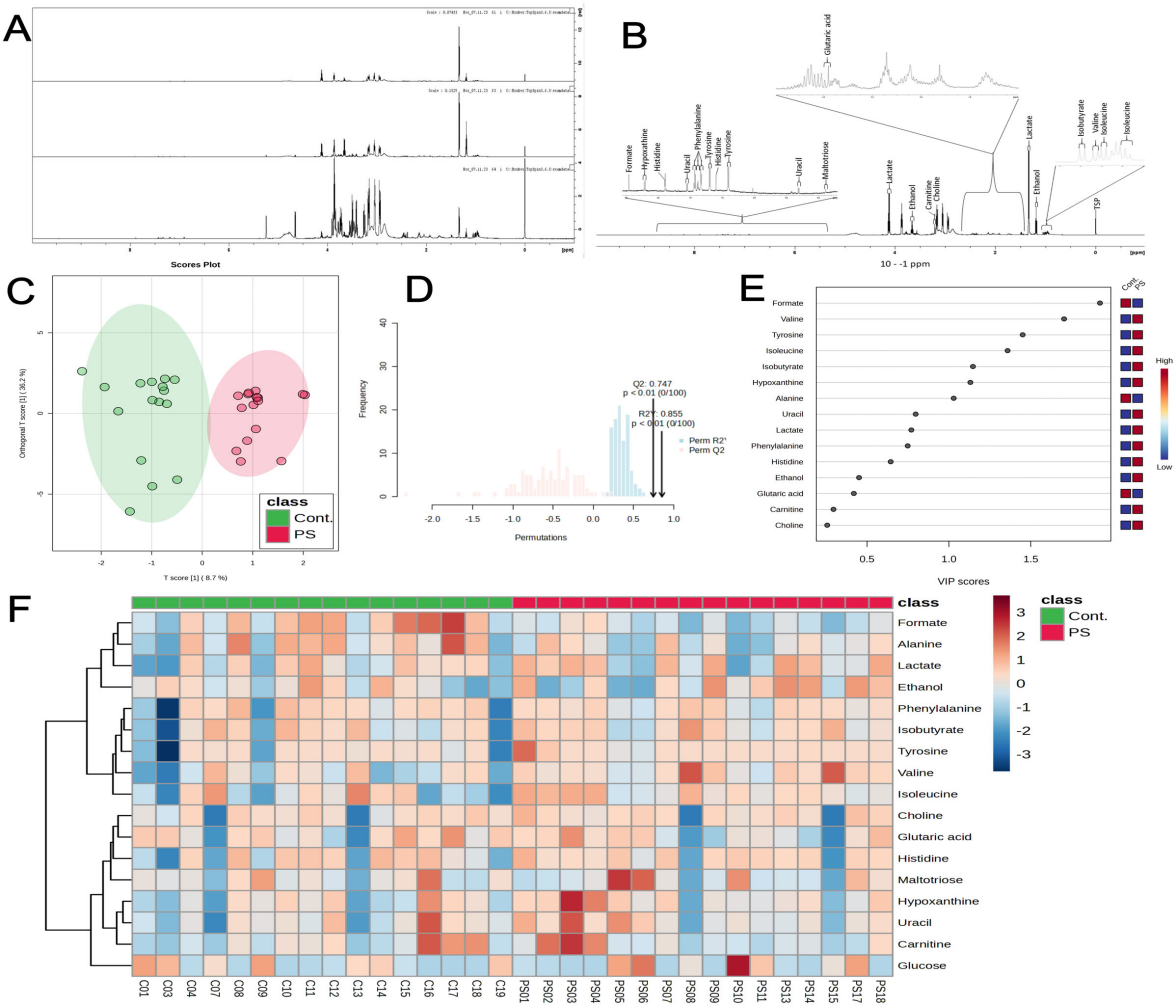
^1^H NMR spectroscopy reveals metabolic alterations in placental chorionic villi explants following 72 h of PS exposure (100 μg/mL). **(A)** Representative ^1^H NMR spectra of control, PS-exposed samples, and pure culture medium. **(B)** List of most prominent metabolites identified in the samples. **(C)** Score plot of orthogonal projections to latent structures discriminant analysis (OPLS-DA), showing the control group (red) and PS-exposed group (green) (Q2 = 0.747, R2Y = 0.855). **(D)** Cross-validation and permutation tests (100 permutations) confirm statistical significance (p < 0.01). **(E)** Variable Importance in Projection (VIP) score. Variables with VIP > 1 are essential for group discrimination, and higher VIP scores indicate more relevant variables in a classification context. The red-blue tiles on the right side of the VIP plot depict the mean expression of each metabolite for the indicated group. **(F)** Heatmap illustrates changes in metabolite concentrations between control (green) and PS-exposed (red) groups. Reddish and blueish tiles indicate increased and reduced concentrations, respectively. All metabolomics supernatant experiments were conducted in triplicates pooled together (n=16).

All supernatant samples underwent analysis using 1H NMR. **Figures 3A, B** depict the metabolite identification and overlapped spectra of PS-exposed and control groups. We employed orthogonal projections to latent structures discriminant analysis (OPLS-DA) for its ability to distinguish predictive from non-predictive (orthogonal) variations. As such, OPLS-DA revealed substantial metabolite differences, separating both clusters (**Figure 3C**). The model exhibited a high degree of separation between groups and good predictive performance through cross-validation (Q2 0.747, R2Y 0.855, and p < 0.01) (**Figure 3D**). The variable importance in the projection (VIP) score highlighted multiple significant alterations, with formate, valine, tyrosine, isoleucine, isobutyrate, hypoxanthine, and alanine being the most important variables (**Figure 3E**). Furthermore, the heatmap visualization of both groups illustrated the similarities among samples within the control or PS groups and the main differences between the analyzed groups (**Figure 3F**), corroborating findings from the correlation plots and OPLS-DA.

**Figure 4:**
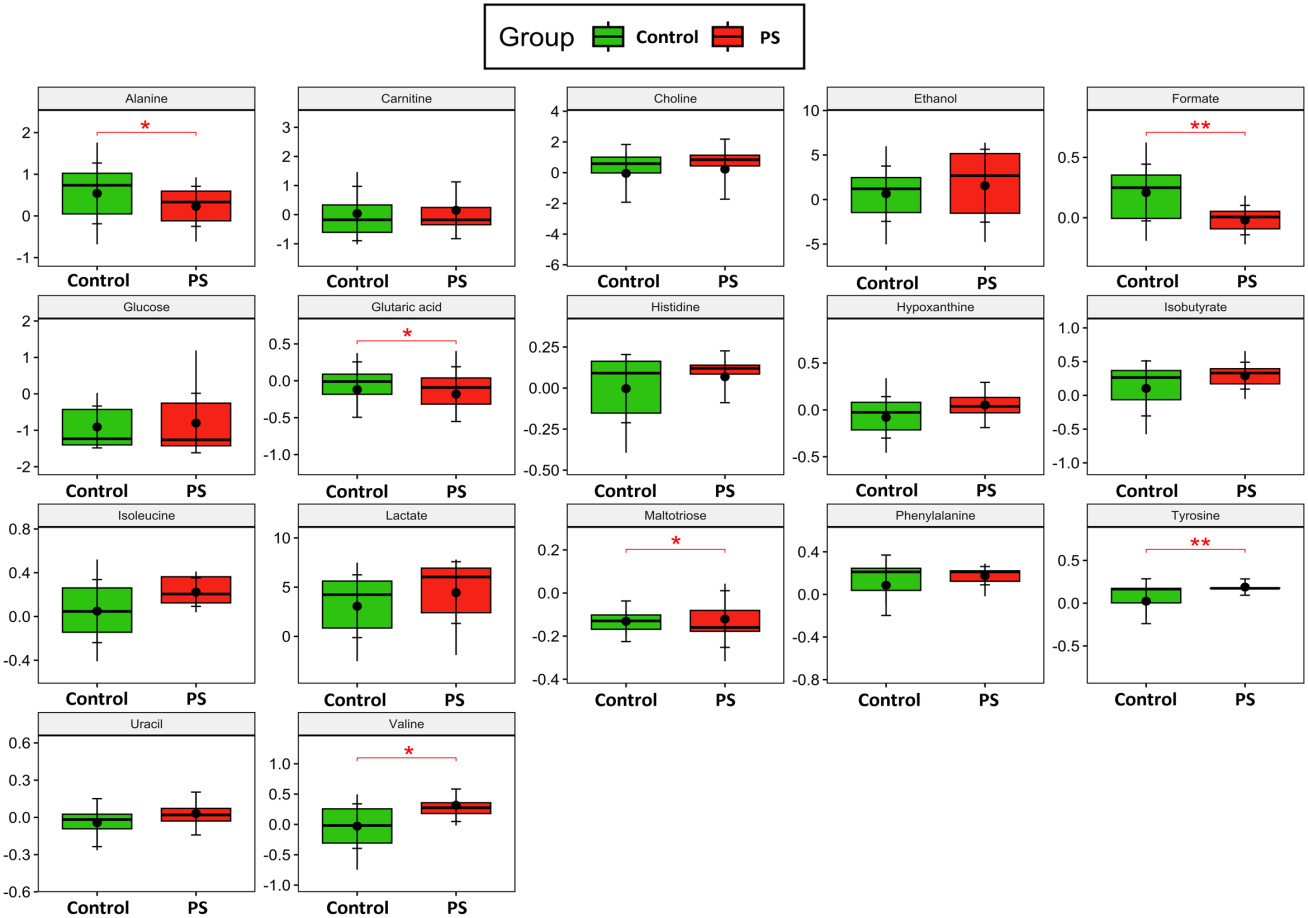
Metabolite Analysis. Box-and-whisker plots depict the relative concentrations of critical variables contributing to the separation of the control (green) and polystyrene (PS) microplastics-exposed (red) groups. Y-axes are represented in relative units, and medians are indicated by horizontal lines within each box, the horizontal brackets and asterisks denote the level of statistical significance for each metabolite. The black dots within each boxplot represents the mean value for each group, while the vertical brackets indicate the standard deviation. All metabolomics supernatant experiments were conducted in triplicates and pooled together (n=16). p values in the respective graphs indicate univariate t-test analysis with FDR correction.

**Figure 5:**
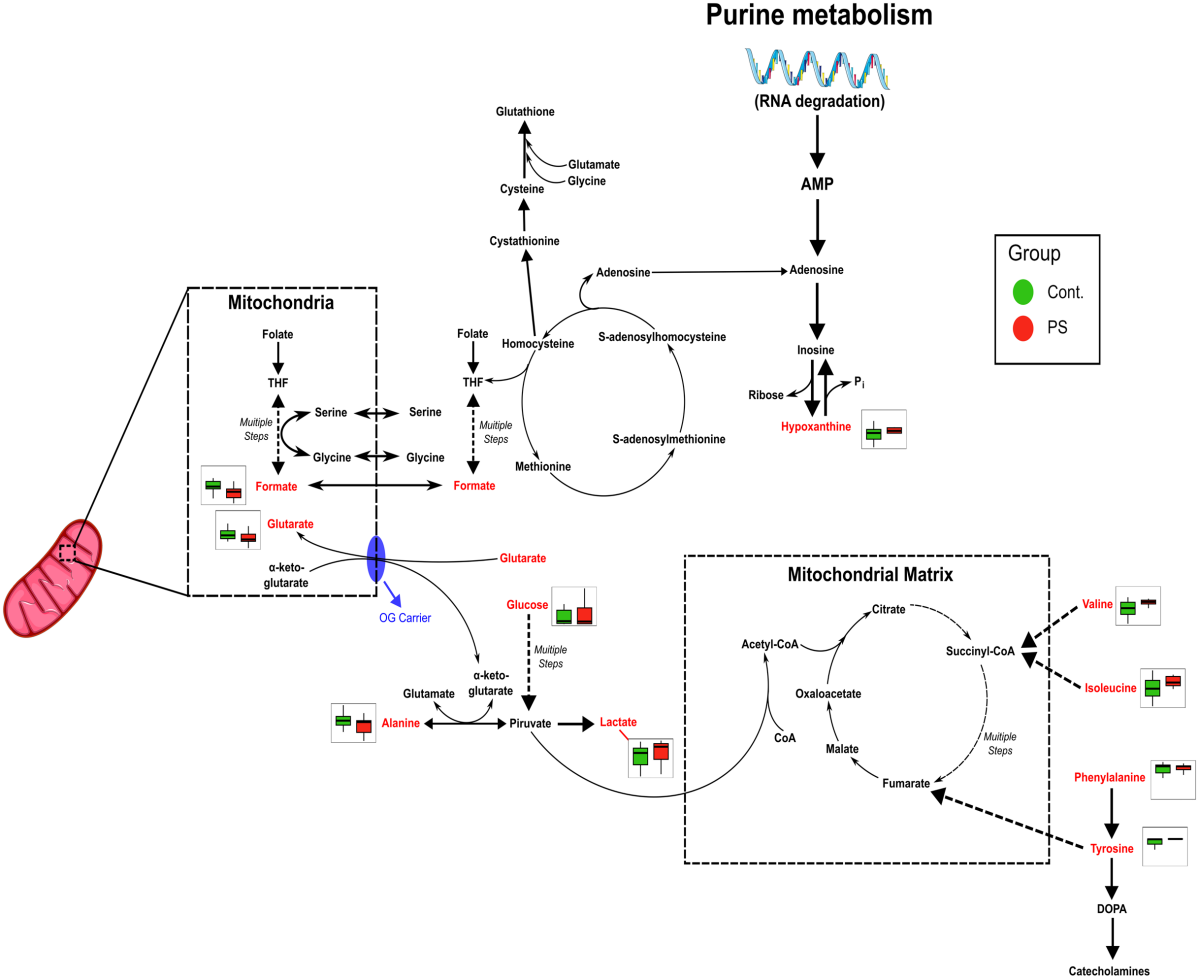
Metabolic pathways affected by PS exposure are inferred from heatmap data, showing alterations in folate metabolism, energy production (TCA cycle) and amino acid metabolism. Pathway impairments suggest potential disruptions in mitochondrial function and energy homeostasis.

The relative concentrations, calculated by normalizing the molar concentration of each metabolite to the total molar concentration of all metabolites for each sample in the two groups, were compared using box-and-whisker plots. The data obtained demonstrated that the PS-MP exposed group exhibited lower levels of alanine (p = 0.03, **Figure 4**), formate (p < 0.001, **Figure 4**), glutaric acid (p = 0.02, **Figure 4**), and maltotriose (p = 0.03, **Figure 4**), whereas showing increase in valine (p = 0.03, **Figure 4**) and tyrosine (p < 0.001, **Figure 4**), confirming the results of previous analyses. These findings suggest potential impairments in folate, and amino acid metabolisms, and alterations in the TCA cycle (**Figure 5**).

Additionally, the observed metabolic changes correlated with oxidative stress alterations found in placental explants (**Supplementary Figure 2**). The heatmaps highlight the Pearson correlation among metabolites and between metabolites and oxidative stress (**Supplementary Figures 2A, B**). Pairwise variable associations were also extracted from a similarity matrix derived from the DIABLO model (**Supplementary Figures 2C, D**). This measure, analogous to a correlation coefficient, estimates the variables’ association relevant to group discrimination. Consequently, the reduced antioxidant system directly correlated with reduced glucose, maltotriose, carnitine, and glutaric acid metabolites, while CAT-reduced levels correlated negatively with lactate levels. The increased GPx, negatively correlated with almost all metabolites (isoleucine, isobutyrate, tyrosine, phenylalanine, lactate, histidine, alanine, carnitine, and formate). Additionally, the reduced GSH/GSSG ratio positively correlated with valine, ethanol, isoleucine, isobutyrate, tyrosine, phenylalanine, lactate, histidine, and alanine reduction. Also noteworthy is the negative correlation between the increase in carbonylated proteins with glutaric acid and hypoxanthine (**Supplementary Figure 2**).

## 4 Discussion

Microplastics have been detected in over 1,300 species, ranging from farm animals and aquatic organisms to birds and insects (57). Evidence of MPs ingestion has been observed in numerous ecosystems, and this trend spans various trophic levels (58). The absorption by the body and tissues translocation can lead to toxicity, physical damage, and chemical harm, including the leaching of toxic additives like endocrine disruptors (59, 60). Moreover, understanding the environmental impacts of MPs is a urgent concern, with growing need to quantify effects within risk assessments (61). Moreover, assessing the environmental impacts of MPs remains challenging due to their variability in polymer size, form, and types, chemical additives, colorants and plasticizers, altogether with different levels of degradation by weathering (57). As such, it is important to highlight the need for detailed MP characterization, relevant controls, and weathering for a correct MP characterization (60). Despite all advancements, several key knowledge gaps remain, with challenges in data comparability, nanoplastics measurements and quantification, and understanding of the mechanisms behind health MP effects (57).

To try to understand MP effects in the human placenta, this study represents a pioneering effort to utilize freshly collected human term placental explants for assessing the impact of MPs on human health, particularly during pregnancy. Our findings reveal that PS-MPs exhibit toxicity that disrupts cellular functions, particularly through oxidative stress. This is consistent with results from other *in vitro* studies involving human cells (38–40), confirming the potential hazards to both maternal and fetal health.

To the best of our knowledge, placental villi explants have not been utilized to investigate the uptake or toxicity of MPs, a fact supported by a review of human *in vitro* and *ex vivo* placental models (41). Despite the debate over the value of term placental explants due to high rates of basal cell death reported (35), several manuscripts state otherwise (43, 62, 63), aligning to our results. It is important to highlight that LDH levels need to be monitored closely because term placental villi explants do indeed lose viability fast *in vitro*. As such, we carefully observed the LDH release in our control groups, finding low variations up to 72 h of culture, which worked in our case, but it is a limitation for studies that need a longer analysis. Despite the limitations, placental explants offer an ethically accepted model with greater complexity than cell line cultures, providing human specificity, a complex mixture of cells and extracellular matrix molecules, and still remain one of the closest experimental models to analyze the functional unit of the placenta within all its complexity.

As such, the explants were exposed to the PS-MPs at various concentrations (0.1-100 µg/mL), since PS is one of the most common polymers found in human placentas (18), and it is one of the most common MP polymers used on experimental studies (38, 64). One limitation in MPs experimental studies is the lack of diversity on commercial polymer types, being the most commonly found and used, PS, PE, and PP. All these polymers are commonly listed among the top five evironmental contaminants and are known to bioaccumulate in living organisms. A plethora of research has been made assessing differential toxicity of these polymers, and mostly data depict that they are toxic in different experimental models, but no polymer-specific toxicity has been described amid them (65–67). Nonetheless, in mice instilled for these polymers, only PS was able to induce pulmonary inflammation (68), and in comparison to other polymers such as PVC and PET, PS seems to be less cytotoxic in HepG2 cells (69).

Herein we employed 5 μm commercially purchased PS-MP, and the size was intentionally selected. Our previous study found that term placentas collected in 2021 in Hawai’i, USA, contained MPs with an average size of 5.14 ± 0.75 μm (19), which is very similar to the commercial MP employed in this study. Besides the size, surface charge and concentration can play a decisive role in the uptake mechanisms and toxicity profile of particles (70). The MP-PS particles used in our study are functionalized with carboxyl groups (-COOH), and they are negatively charged, with a zeta potential of -1.67 mV (**Supplementary Figure 3**). It is known that -COOH groups acquire negative charges in aqueous environments due to weathering, thus influencing the behavior and interaction with other pollutants in the environment (71, 72), which was also one feature strategically chosen to better mimic the chemical surface type of a PS-MP in the environment, slightly reducing the gap between pristine and weathered MPs. Nonetheless, it is important to highlight that the MP used was pristine, completely round, and assumed to not have chemical additives, which are characteristics not similar to weathered MPs.

Regarding the chosen concentration, it is impossible to know for sure which concentration of MP would be the most similar to concentrations found in pregnant women. No large epidemiological studies exist to determine the MPs average concentration in the blood, even less studies on pregnant women. The closest we have is one study that estimated the total amount of MPs in the blood as 1.6 µg/mL, from 22 Dutch volunteers (29), but we still need robust epidemiological studies to access the real concentration of MPs in the worldwide population, and using more than one technique to characterize these MPs. Moreover, we currently lack knowledge regarding the precise daily absorption of microplastics, its bioaccumulation rate in different organs and their resistance to degradation and elimination from the body (40). The concentration of MPs in human placentas is also debatable, with a recent study showing up to 126.8 ± 147.5 µg/g of MPs in 10 placentas from the USA (23), which would even align with the high *in vitro* concentration used herein. Nevertheless, we could have chosen 1 or 10 µg/mL concentrations to this study, but we were initially intrigued by that tendency increase in the 24 h MTT assay, followed by the time-dependent cytotoxicity observed in the LDH release assay after 72 h of exposure. Therefore, we decided to investigate the mechanisms involved using 100 µg/mL of PS-MP. The chosen concentration was also consistent with those reported in previous studies investigating transplacental transport, which used similar concentrations, and even exceeded, using up to 1,000 μg/mL of PS-MP in cell lines (41).

The observed increase in cytotoxicity confirms the findings from several studies demonstrating PS-MNP cytotoxicity in pregnant mice and their offspring (73, 74), placental cell lines (38, 39), and embryo stem cells (44). This result was different from two previous studies that reported that only positively charged PS-MNPs were able to induce cytotoxicity, whereas negatively charged ones not (75, 76). It is worth noting that alterations in the steric shielding of the charge on a surface moiety, resulting from subtle distinctions in the chemical structure of the charge-bearing moiety, might affect the cellular uptake and toxicity of the MNPs (77).

In addition to increased cytotoxicity, PS-MP exposure induced humungous levels of reactive oxygen species (ROS), such as O_2_^•–^ and H_2_O_2_. Antioxidant enzyme levels were mostly reduced, with lower SH content, diminished CAT and SOD activity, and a decreased GSH/GSSG ratio. The evident oxidative stress resulted in elevated levels of MDA and carbonyl proteins, suggesting substantial damage to lipids and proteins due to the heightened levels of ROS. Our findings regarding cytotoxicity align with previous reports in the literature, which extensively document the induction of oxidative stress following exposure to PS-MPs (78–80). Additionally, increased production of ROS, oxidative stress, and mitochondrial dysfunction have been consistently reported in various human cell types (81–84). In this context, the results of this study support the idea that the long-term exposure of plastic particles produces higher levels of ROS, which could improve toxicity and cause other adverse pregnancy outcomes (40).

Not only did PS-MP increased cytotoxicity, oxidative stress, protein carbonylation, and lipid peroxidation, but they affected the entire placental metabolism.The disrupted amino acid levels may indicate disturbances in protein metabolism, while the altered levels of hypoxanthine and formate correlate directly with deficiencies in folate and purinergic metabolism. Collectively, these changes are associated with oxidative stress responses and mitochondrial dysfunction, which contribute to metabolic imbalances Oxidative stress can impair the enzymes involved in gluconeogenesis, TCA cycle, amino acid catabolism, and energetic metabolism (85), which are known to change the metabolites previously described. Such changes are also present in important gestational diseases, such as preeclampsia and gestational diabetes mellitus (86). The oxidative stress and the metabolic changes can limit the supply of essential nutrients to the fetus, leading to intrauterine growth restriction, stillbirth, preterm birth, neurodevelopmental disorders, and postnatal health issues (87, 88). Similar results in PS-MNP exposure of intestinal cell lines, with oxidative stress, increased glycolysis, disrupted energy, and glutamine metabolism changes have been described (89). Perturbations in biotin metabolism, lysine degradation, and glycolysis/gluconeogenesis pathways in pregnant mice exposed to PS-MNP have also been described (90). In other studies, the researchers found changes in the lipids metabolism in different cell lines (91, 92), in addition to studies that demonstrated disruptions of glucose, energy, and lipid metabolisms in zebrafish and eels (93, 94). Additionally, Li and collaborators (95), reported disruptions of redox homeostasis, carbohydrate, and energy metabolism in barley, indicating solid and preserved alterations of this pathways across different types of experimental models. Therefore, it is essential to highlight that our results indeed demonstrate that PS-MPs are harmful to human placentas, altering key biochemical mechanisms that can reverberate to other molecular effects and pathways, which might be detrimental to fetal development, in complete consonance to what has been observed in wild and experimental animals.

Mechanistically, the oxidative stress and the metabolic changes observed herein seem to be happening mostly due to a mitochondrial dysfunction, although we need further research to prove this hypothesis in our model. The mitochondria, as a central hub of cellular energy metabolism and redox balance, are especially susceptible to environmental stressors. The MPs cause NADPH oxidase 4 (NOX4)-mediated mitochondrial dysfunction, as demonstrated by membrane potential changes, impaired cellular energy metabolism, and repression of mitochondrial respiration in different cell models (96, 97). A review compiled all the mitochondrial alterations caused by MPs exposition: disruption of mitochondrial membrane potential, induction of mitochondrial depolarization, mitochondrial damage, mtDNA damage, and mitochondrial oxidative stress, leading to the conclusion that MPs can indeed be involved in mitochondrial dysfunction (98). Possibly caused by this mitochondrial dysfunction, the remarkable oxidative stress we found is leading to oxidative damage to cellular components, including lipid peroxidation and protein carbonylation. Other interesting evidences of mitochondrial dysfunction are the altered TCA cycle and amino acid metabolism. Studies have shown that MP exposure disrupts these metabolic pathways, leading to a shift toward glycolysis as an alternative energy source, while disruptions in amino acid metabolism, indicate alterations in protein synthesis and degradation (99, 100).

In summary, this study indicates that PS-MP exposure induces time-dependent cytotoxicity, oxidative stress, and metabolic alterations in placental explants, highlighting potential risks for maternal and fetal health. The results highlight the immediate risks associated with MPs exposure, highlighting an urgent need for targeted resources and strategic initiatives to address plastic pollution at the environmental level, since it is where humans are being exposed. This includes the exploration of new degradation strategies for the effective removal of MP, such as biodegradation, advanced oxidation degradation, and photocatalytic degradation (40). Given the substantial implications of these findings, it is paramount to expand our research efforts, elucidating the ramifications of varied MP exposures on both pregnancy and fetal development, as well as the comprehensive effects of MP contamination on subsequent generations. Particularly, which other effects PS-MP and other polymers can cause in the placenta, and how these changes would affect fetal development, including epigenetic alterations. By demonstrating the impact of PS-MP on placental tissues, this study contributes to the growing body of evidence that underscores the need for comprehensive public health strategies and policies to mitigate the effects of plastic pollution. Moving forward, future research should concentrate on the comparative toxicity of PS-MPs with other prevalent polymers, and chemical mixtures, to better understand the relative risks to human pregnancy.

## Data Availability

The raw data supporting the conclusions of this article will be made available by the authors, without undue reservation.
