# Supplementary material for: Polystyrene microplastics exposition on human placental explants induces time-dependent cytotoxicity, oxidative stress and metabolic alterations
Source: Front Endocrinol (Lausanne). 2024 Nov 20;15:1481014. doi: 10.3389/fendo.2024.1481014 (PMC11614646; doi:10.3389/fendo.2024.1481014)
Supplement: Supplementary file 1 [file DataSheet1.pdf]

Supplementary Material

1 Supplementary Figures

ID	Name	Spectrum	Chemical Structure	HQI
90	Polystyrene 2. Clear Sphere			59.03

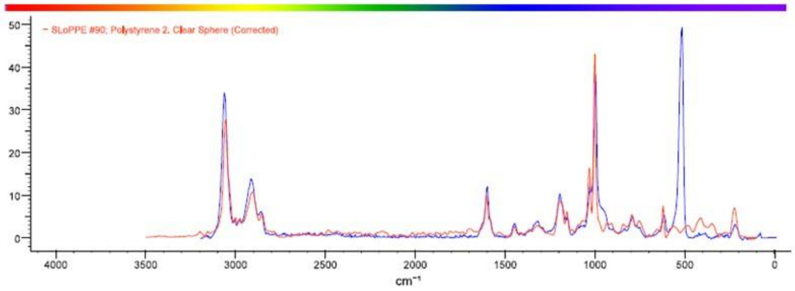

Name	Value
Name	Polystyrene 2. Clear Sphere
Accumulation	10
Acquisition Time	100 s
Date	01.02.2019 13:29
Delay time (s)	0 s
Detector Name	Syncerity OE
Filter	25%
Grating	1200 (750nm)
Hole (µm)	300
Instrument Name	XploRA Plus
Laser (nm)	532nm_Edge
Objective	x100_VIS_LWD
Resolution	3.252 cm-1/pixel
Sample Name	PS 2

Database/Hit List Structure

Supplementary Figure 1. Raman spectroscopy of the purchased polystyrene microplastic beads.

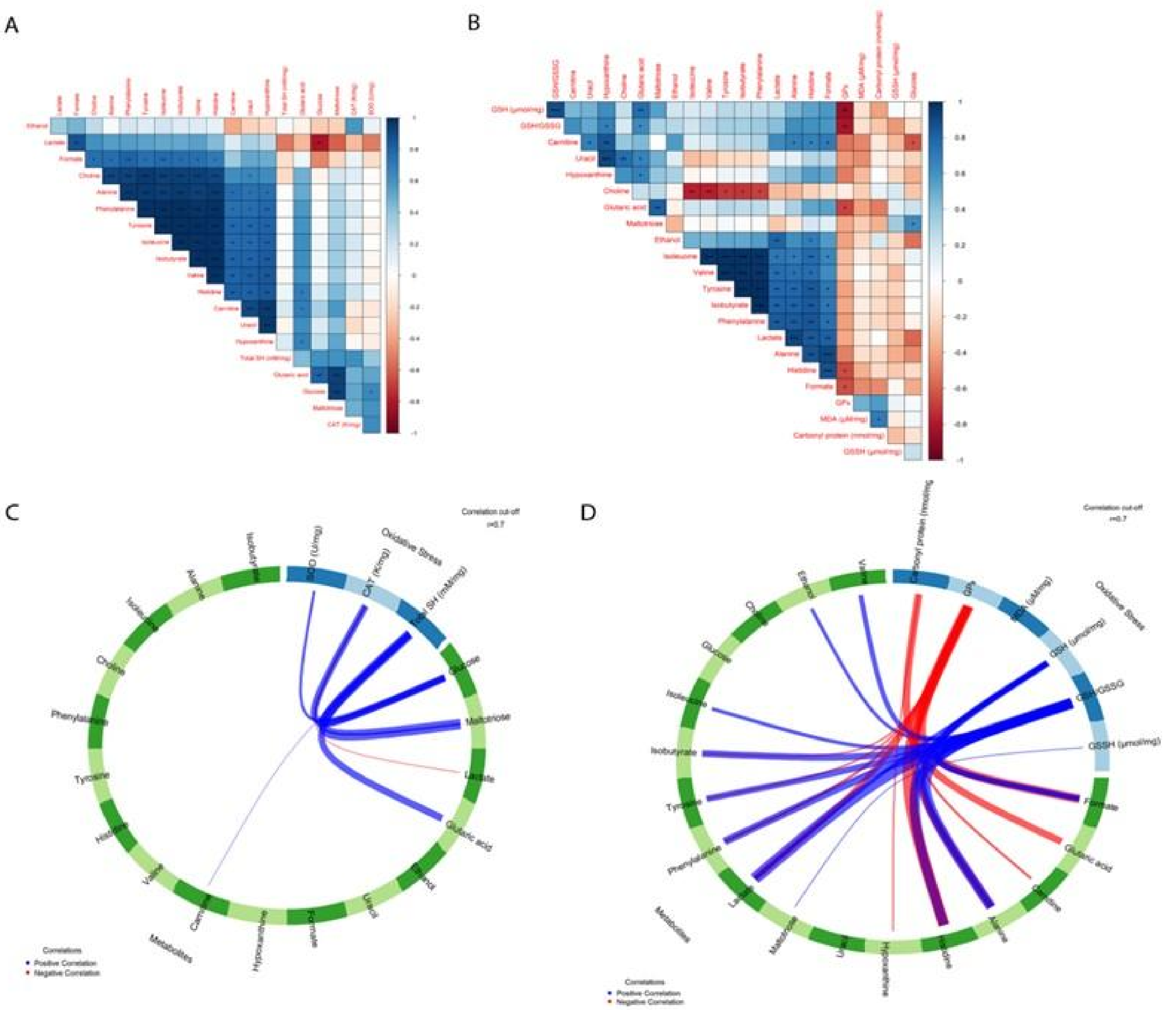

Supplementary Figure 2: Correlation of Metabolic Changes with Oxidative Stress Alterations in Placental Explants. To assess the association between metabolic changes and oxidative stress alterations in placental explants, the Pearson correlation coefficient and a multiblock Partial Least Squares Discriminant Analysis (PLS-DA) model, specifically known as Data Integration Analysis for Biomarker Discovery using Latent Components (DIABLO), were employed. (A-B) Heatmaps illustrating the Pearson correlation among metabolites and between metabolites and oxidative stress. The heatmaps are divided to precisely match the samples used for all techniques. Red and blue tiles indicate positive and negative correlation, respectively. $*p < 0.05$, $**p < 0.01$, $**p < 0.001$. (C-D) Pairwise variable associations extracted from a similarity matrix derived from the DIABLO model. Blue lines indicate positive correlations, and red lines indicate negative correlations. Metabolites are depicted in green tones, while oxidative stress markers are shown in blue tones.

			Mean (mV)	Area (%)	St Dev (mV)
Zeta Potential (mV):	-1,67	Peak 1:	-1,67	100,0	3,53
Zeta Deviation (mV):	3,53	Peak 2:	0,00	0,0	0,00
Conductivity (mS/cm):	1,81	Peak 3:	0,00	0,0	0,00
Result quality	Good				

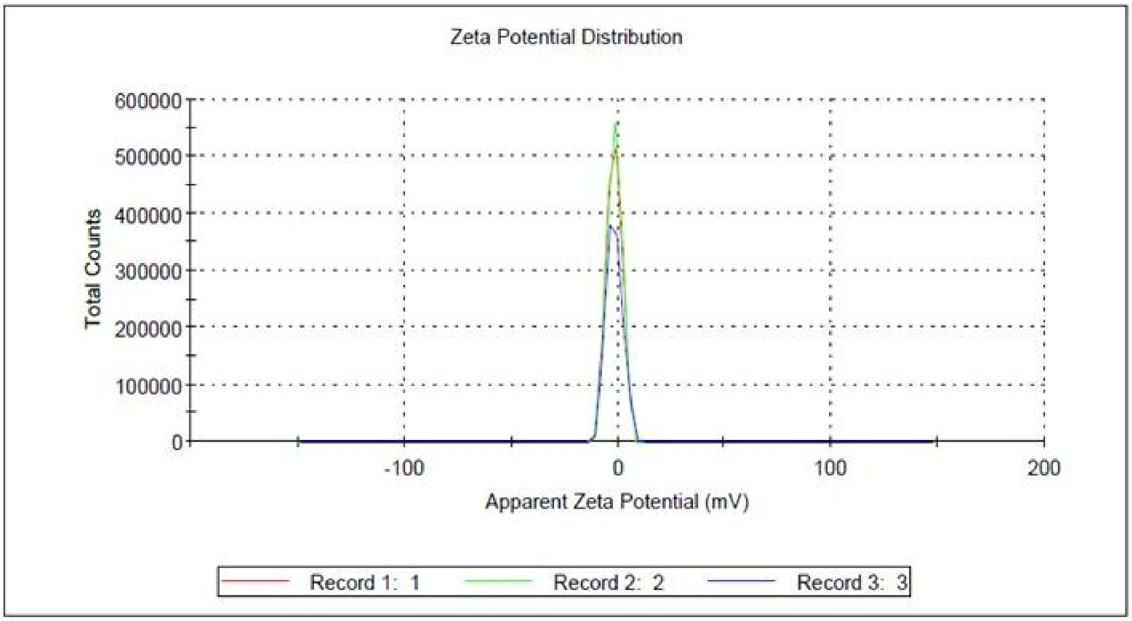

Supplementary Figure 3: Zeta potential of 100 µg/mL of the comercial PS-MP in DMEM/F12 culture medium.
